# Coal–rock damage characteristics caused by blasting within a reverse fault and its resultant effects on coal and gas outburst

**DOI:** 10.1038/s41598-021-98581-w

**Published:** 2021-09-27

**Authors:** Kui Gao, Ping Huang, Zegong Liu, Jian Liu, ChiMin Shu, Guodong Qiao

**Affiliations:** 1 School of Safety Science and Engineering, Anhui University of Science and Technology, Huainan, 232001 Anhui China; 2 Key Laboratory of Mine Safety and High Efficient Mining Jointly Built By Province and Education Ministry, Anhui University of Science and Technology, Huainan, 232001 Anhui China; 3 School of Environment and Resources, Fuzhou University, Fuzhou, 350108 Fujian China; 4 Department of Safety, Health, and Environmental Engineering, National Yunlin University of Science and Technology, Yunlin, 64002 Taiwan, ROC

**Keywords:** Petrology, Structural geology

## Abstract

In view of the coal and gas outburst accidents occur frequently caused by blasting in geological structural belt, in order to study the mechanical characteristics of coal rock in tectonic belt disturbance by blasting and blasting vibration effect influenced on the stability of surrounding rock, coal–rock damage and failure characteristics within a reverse fault caused by a blasting stress wave were investigated using numerical analyses and experiments. According to the experimental results, the causes of coal and gas outburst dynamic disasters within a reverse fault during blasting are analyzed. The outcomes indicated that the crushing circle created by the crack propagation near the blasting hole severely damaged the fault plane and floor rocks adjacent to the footwall of the reverse fault. Fractures also extended to the upper and lower coal seams of the reverse fault; this caused the surface of the coal seam to fall off and severe internal damage. According to theoretical analysis, the reflection of the blasting stress wave propagating to the reverse fault intensified the damage to coal and rock. Elastic strain energy accumulation within the reverse fault structural zone was accompanied by high-stress concentration. The reverse fault tectonic region was destroyed by blasting vibration. It increased gas pressure and caused a weak surface, which provided a channel for gas flow and a dynamic basis for the occurrence of coal and gas outburst. The research results have important theoretical value to reveal the mechanism of coal and gas outburst in tectonic belt induced by blasting.

## Introduction

Coal and gas outburst is a nonlinear dynamic process of energy accumulation and unsteady energy release of coal, rock and gas system in coal mine^1^. In general, although these disasters only last for a few seconds or tens of seconds from initiation to termination^2^, hundreds or even tens of thousands of tons of coal can be ejected from the wall onto adjacent roadways with large volumes of discharged gases^3^. These outbursts mainly occur in local areas within mined coal seams, which are generally adjacent to a structural zone^4,5^. Before the 1950s, the research on coal and gas outburst was mainly field investigation and data analysis. Since the 1950s, many scholars began to use the method of laboratory test to study coal and gas outburst, achieved rich results and accumulated valuable experience^6,7^.

With an increase in mining activities, controlled blasting technology has been increasingly used in mining technologies to enhance seam permeability^8,9^, to cause forced hard roof caving^10,11^, and to weaken the top of hard coal in thick seams. These effects improve the mining rate and gas extraction efficiency and speed up tunneling into hard rock through deep hole blasting^12^. However, in blasting applications, a stress wave can disturb a structural area and readily induce coal–rock and gas dynamic disasters. Some statistics regarding coal–rock and gas dynamic disasters due to blasting are listed in Table 1.

**Table 1:** Selected coal and gas dynamic disasters caused by blasting: summarised statistics in China.

Time	Name of mine	Causes of outburst	Coal seam characteristics	Outburst coal (rock) quantity (t)	Outburst gas quantity (10,000 m^3^)	Number of deaths (persons)
2014.01.04	Zhongtian Coal Mine, Guizhou Province	Blasting excavation	Coal bed thickening	260	2.4	4
2014.06.11	Xinhua Coal Mine, Guizhou Province	Coal uncovered by blasting	Soft coal seam	1010	12	10
2015.08.11	Zhengzhong Coal Mine, Guizhou Province	Blasting	Outburst area	218	2.8	13
2016.03.06	Songshu Town Coal Mine, Jilin Province	Blasting excavation	Graben structure	NA	NA	12
2017.01.04	Xingyu Coal Mine, Henan Province	Blasting excavation	Outburst coal seam	NA	NA	12
2018.04.04	Heilongjiang Didaoshenghe Coal Mine, Heilongjiang Province	Blasting excavation	Graben structure	67	0.23	5

Previous studies have made statistics on the relationship between blasting and coal and gas outburst. The results show that there is a direct correlation between coal and gas outburst and blasting, and most gas outbursts are caused by blasting^13^; The influence of geological structure and blasting technology on coal and gas outburst is analyzed, but the article on coal and gas outburst simulation experiment under the joint action of geological structure and blasting is rare^14^. Limited studies have been conducted on the relationship between blasting and coal and gas outbursts^15^. To date, no in-depth study has been performed on the damage and failure characteristics of coal within structural areas under explosion loads. This study aimed to optimise control blasting technology and prevent the occurrence of dynamic gas disasters by understanding the physical processes caused by these activities.

This study also investigated the damage and failure characteristics of coal and rock and their influence on outburst dynamical disasters by combining numerical simulation, similarity experiments, and theoretical analyses to address the problem of coal and rock dynamic evolution within reverse fault tectonic belts under the action of blasting stress waves.

## Blast disturbance through numerical simulation

### Model construction

**Figure 1:**
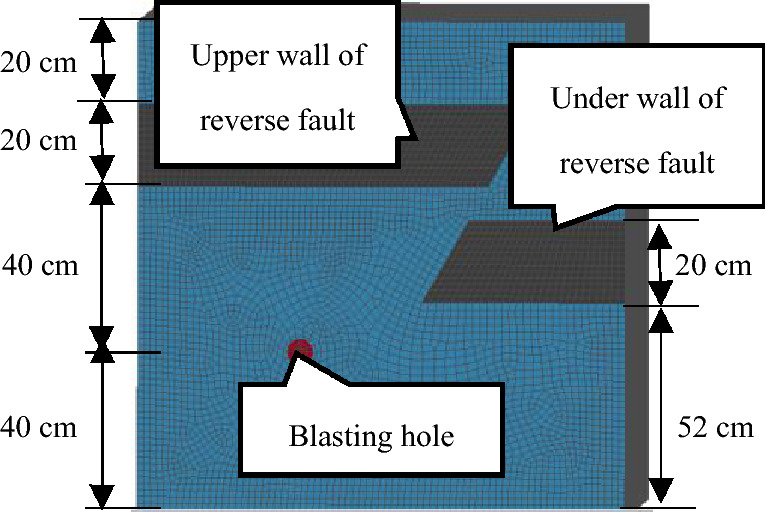
Three-dimensional (3-D) numerical blasting model used in this analysis.

DYNA^3-D^ was used for numerical simulations through a three-dimensional (3-D) numerical model (Fig. 1). The length, width, and height of this numerical model were 1.2 m each. Blasting holes were arranged in the lower rock strata 40 cm from the upper wall of the reverse fault, and the thickness of the coal seam was 20 cm. The soft coal and rock mechanical parameters within the reverse fault are summarised in Table 2.

**Table 2:** Tectonic coal and rock parameters used in numerical simulation.

Blasting medium	Density (g/cm^3^)	Modulus of elasticity (GPa)	Poisson’s ratio	Yield strength (MPa)
Tectonic coal	1.4	5.3	0.32	6.9
Rock	2.4	9.8	0.24	35

The detonation pressure acting on coal and rock at any given time can be expressed as follows:

1 $$P = A\left( {1 - \frac{\omega }{{R_{1} V}}} \right)e^{{ - R_{1} V}} + B\left( {1 - \frac{\omega }{{R_{2} V}}} \right)e^{{ - R_{2} V}} + \frac{{\omega E_{0} }}{V},$$

where *P* is the pressure of the blasting product, MPa; *A* and *B* are the parameters of explosives, GPa; *V* is the relative volume of detonation products, m^3^; *E*_0_ denotes the internal energy generated by detonation products, GPa; and *R*_1_, *R*_2_, and *ω* are characteristic dimensionless explosive parameters^16^. The explosive parameter settings used in numerical calculations are presented in Table 3. Post explosive load action, the failure forms observed in coal and rock mainly included tensile stress and compressive shear. Coal and rock fractures under pressure (*P*) can be expressed as follows:

2 $$\left\{ \begin{gathered} P \le P_{\max } \hfill \\ P \ge P_{{{\text{min}}}} . \hfill \\ \end{gathered} \right.$$

**Table 3:** Explosive parameter settings used in the numerical model.

Density (g/cm^3^)	Detonation velocity (m/s)	*A* (GPa)	*B* (GPa)	*R*_1_	*R*_2_	*ω*	*E*_0_ (GPa)
0.95	3,200	347	0.733	4.15	0.95	0.3	1.0

In these expressions, *P*_max_ and *P*_min_ denote maximum compressive and minimum tensile strengths of coal and rock, respectively. Thus, when *P*_max_ is negative, *P*_min_ is positive (both expressed in MPa).

### Analytical results

**Figure 2:**
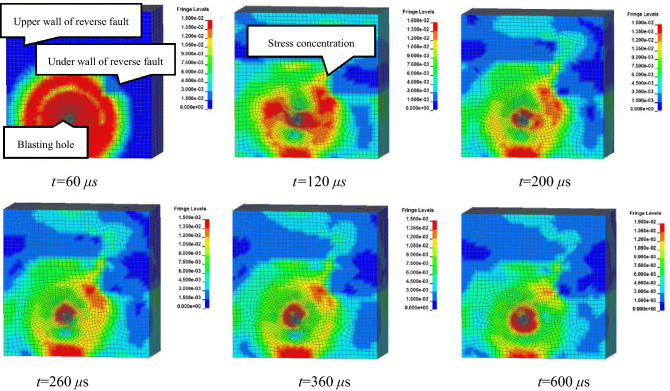
Part contours of 3-D effective stress at different times. (1) pressure gauge mounting hole; (2) pressure conduction device; (3) gas hole; (4) box wall; (5) hydraulic jackhole; (6) pressure transmitting steel plate; (7) bolt hole; (8) front cover plate; (9) blasting wireway lead hole; (10) back cover plate; (11) strain brick wireway lead hole.

Effective stress at different moments along the cut surface of a blasting hole is displayed in Fig. 2. These simulations revealed that when t = 60 μs, a blasting stress wave propagated uniformly along the hole at the initial time of development, and in this case, stress was less affected by the reverse fault. However, when t = 120 μs, the propagation of a stress wave was prominently influenced by soft coal seams within the reverse fault. Blasting stresses on the coal–rock interface adjacent to the reverse fault plane and coal seam near the hole were considerably greater than those on the other side, which led to the concentration of blasting stress. Throughout this process, a soft coal seam was subjected to the blasting stress to a lesser extent than within the rock seam. Blasting stress concentration mainly occurred near the hole and the area adjacent to the fault plane at the footwall of the reverse fault after 120 μs.

These numerical simulation results revealed that blasting stress wave propagation was considerably influenced by the reverse fault. Furthermore, coal and rock within the reverse fault under the wall adjacent to the hole were most strongly disturbed by blasting stress.

## Experimental blast disturbance validation

An experimental model was established using similarity theory and numerical simulation results. This simulation experiment was conducted in the laboratory to reproduce the damage and failure characteristics of a blasting stress wave on a reverse fault structural belt^17,18^.

### Similarity theory

The Froude proportional method was used to establish the experimental model used here^19^, which satisfied the following scale factor:

3 $$K_{\sigma } = K_{\rho } K_{l} .$$

Thus, we obtain the following expression:

4 $$\left\{ \begin{gathered} K_{\sigma } = \frac{{\sigma_{m} }}{{\sigma_{p} }} \hfill \\ K_{l} = \frac{{l_{m} }}{{l_{p} }} \hfill \\ K_{\uprho } = \frac{{\rho_{m} }}{{\rho_{p} }}. \hfill \\ \end{gathered} \right.$$

In these expressions, *K*_σ_, *K*_ρ_, and *K*_l_ represent proportional coefficients of similar stress, density, and length, respectively. The basic variables used are length, *l,* and density, *ρ*, whereas *m* and *p* refer to the model and prototype, respectively.

Thus, *K*_ρ_ ∈ (1.24, 1.6), *K*_σ_ ∈ (0.15, 0.4), and *K*_l_ = *K*_σ_/*K*_ρ_ = (0.15/1.6, 0.4/1.24) = (0.09, 0.32). The sample used in this experiment was tectonic soft coal; thus, on the basis of the stress ratio range, *K*_σ_ is 0.4 and *K*_ρ_ is 1.6. Thus, *K*_l_ = *K*_σ_/*K*_ρ_ = 0.25.

The similarity relationship between explosion load and charge amount can, therefore, be calculated as follows^19^:

5 $$\left\{ \begin{gathered} K_{\sigma } = K_{{\text{f}}} K_{\rho } K_{{\text{C}}} K_{{{\overline{\text{R}}}}}^{ - n} \hfill \\ K_{{{\overline{\text{R}}}}} = \left( {\frac{{K_{\sigma } }}{{K_{{\text{f}}} K_{\rho } K_{{\text{C}}} }}} \right)^{{ - \frac{1}{n}}} . \hfill \\ \end{gathered} \right.$$

Thus, the proportional distance between experimental model material and coal in the field can be expressed as follows:

6 $$\left\{ \begin{gathered} \left( {\frac{R}{{W^{1/3} }}} \right)_{{\text{p}}} = K_{{{\overline{\text{R}}}}} \left( {\frac{R}{{W^{1/3} }}} \right)_{{\text{m}}} \hfill \\ \frac{{(W^{1/3} )_{{\text{p}}} }}{{(W^{1/3} )_{{\text{m}}} }} = \frac{1}{{K_{{{\overline{\text{R}}}}} }}\frac{{(R)_{{\text{p}}} }}{{(R)_{{\text{m}}} }} \hfill \\ K_{{{\text{W}}^{1/3} }} = \frac{{K_{{\text{L}}} }}{{K_{{{\overline{\text{R}}}}} }} \hfill \\ C = \sqrt {\frac{E}{\rho }} . \hfill \\ \end{gathered} \right.$$

In this expression, *W* refers to the number of explosives loaded, *C* represents the stress wave velocity, *n* expresses the attenuation coefficient, *f* annotates the coupling coefficient, $\overline{R}$ stands for proportional distance such that $\overline{R}$ = *R*/*W*^1/3^, and *R* symbolizes the blasting centre distance.

Because some energy leaks from coal during cylindrical charge presplit blasting, the proportional coupling coefficient, *K*_f_ = 2.0; the proportional stress wave coefficient, *K*_c_ = 0.41; and the attenuation coefficient, *n* = 2.5, are all recorded. Therefore, the proportional distance coefficient can be expressed as:

7 $$K_{{{\overline{\text{R}}}}} = 1.6.$$

Thus, if a 100-mm columnar second-level permissible water glue explosive charge is present within a mine, 25 mm is required for this experiment.

### Simulated experimental device

**Figure 3:**
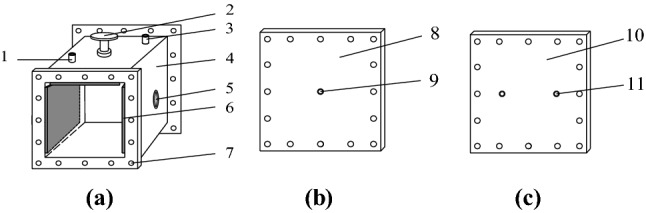
An experimental device used for blasting simulations: (**a**) main body of the box, (**b**) front cover plate, and (**c**) rear cover plate.

**Figure 4:**
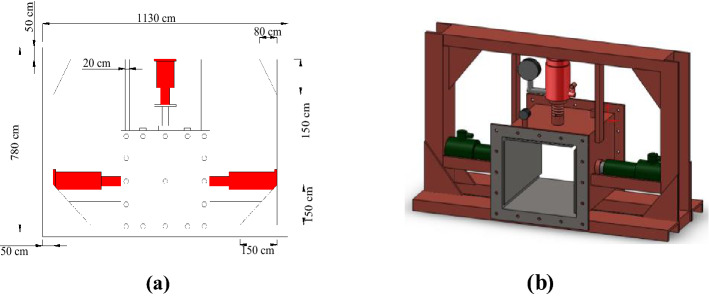
Schematic of the experimental blasting device used in this study: (**a**) device main view, and (**b**) 3-D schematic diagram.

A special gas–solid coupling blasting simulation test system was used for all experiments. The size of the inner cavity of the experimental chamber is 30 cm × 30 cm × 30 cm (Fig. 3). The experimental chamber consists of main body of the box (Fig. 3a), front cover plate (Fig. 3b) and rear cover plate (Fig. 3c). The connection between the loading device and box was sealed with a ring, and the front and rear sides were sealed with high strength silica gel pads and bolts. The loading device used in this study comprised three hydraulic jacks and transfer plates (Fig. 4). Where Fig. 4a is the experimental blasting device main view and Fig. 4b is the 3-D schematic diagram.

### Experimental data monitoring system

**Figure 5:**
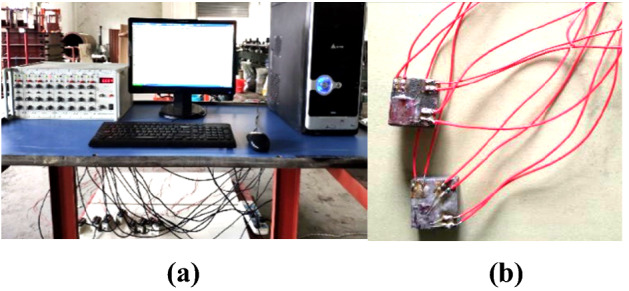
Experimental data monitoring system and strain brick used in this study: (**a**) test data monitoring system, and (**b**) strain brick.

An SDY2107A super dynamic strain data acquisition system was used in this analysis to investigate stress values in three directions from the bricks during the blasting process. The experimental data monitoring system (Fig. 5a) and strain brick used in the experiments are illustrated in (Fig. 5b).

In a plane stress model based on strain values, corresponding stress values for measuring points can be calculated as follows:

8 $$\sigma = \frac{E}{2(1 - \mu )}(\varepsilon_{1} - \varepsilon_{3} ) \pm \frac{E}{2(1 + \mu )}\sqrt {(\varepsilon_{1} - \varepsilon_{3} )^{2} + (2\varepsilon_{2} - \varepsilon_{1} - \varepsilon_{3} )^{2} } .$$

In this expression, *ε*_1_ refers to the horizontal strain, *ε*_2_ expresses the strain at 45°, *ε*_3_ annotates the vertical strain, and *E* denotes the elastic modulus, GPa.

Ultrasonic information can reflect changes in microdamage and cracks in coal and rocks. The wave velocity of an ultrasonic wave is related to the elastic modulus, density, and Poisson’s ratio of coal and rock samples. Thus, under explosive load action, the propagation of acoustic waves results in diffraction, reflection, and scattering, causing a decrease in the overall propagation speed.

**Figure 6:**
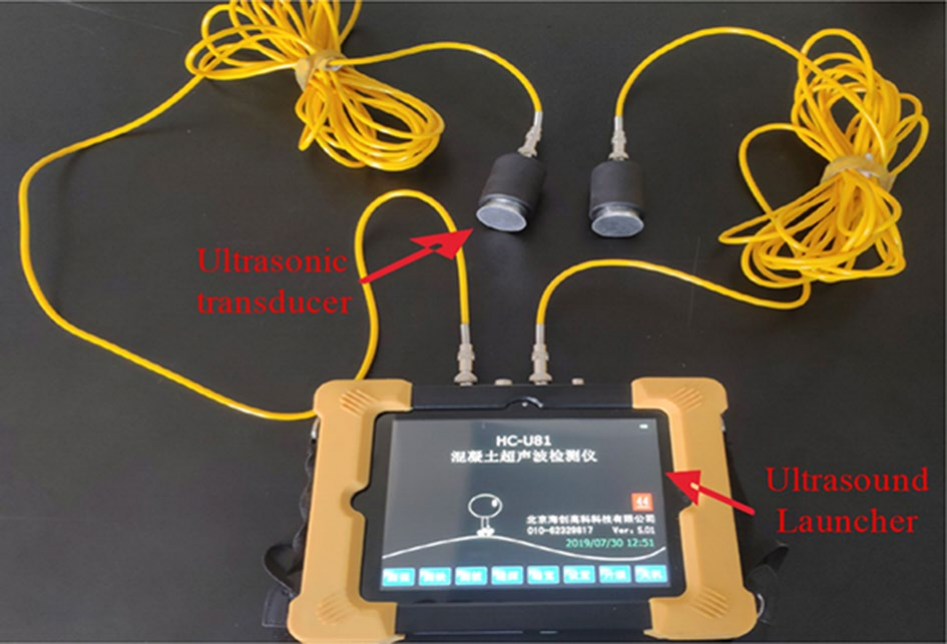
Ultrasonic wave velocity testing system used in this study.

A U-81 concrete ultrasonic detector developed by the Beijing Haichuang Hi-tech Technology Co. (Beijing, PR China) was used in this study (Fig. 6).

### Experimental cartridge preparation

**Figure 7:**
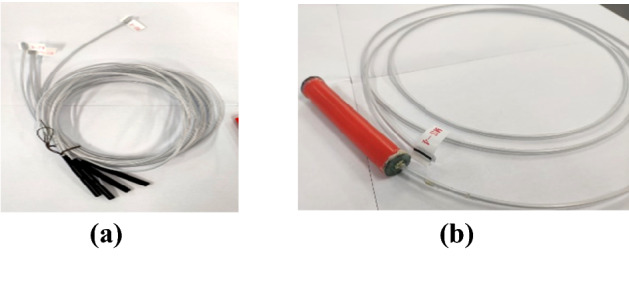
Experimental cartridge production system with (**a**) detonator, and (**b**) blasting cartridge.

The experimental cartridge used here was prepared from a polyvinyl chloride tube with a diameter and thickness of 16.0 and 1.0 mm, respectively (Fig. 7). Second-level permissible water glue explosives used in coal mines were filled into the cartridge during the experiment, and a special explosive booster of the same length was placed in the cartridge tube. This detonator was used to detonate the explosive (Fig. 7a).The finished blasting cartridge is shown in (Fig. 7b).

### Experimental model construction

**Figure 8:**
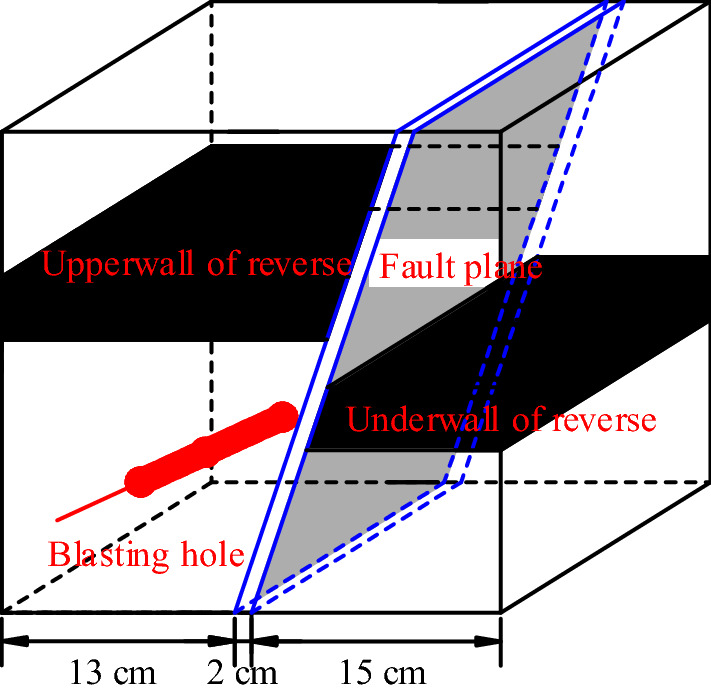
Experimental blasting test model.

The experimental model used in this study measured 30 cm × 30 cm × 30 cm. This meant that applying the length similarity ratio, field coal, and rock of 1.2 m × 1.2 m × 1.2 m dimensions can be simulated. Blasting holes were arranged in the rock layer 10 cm away from the upper wall of the fault, and the thickness of the coal seam was 5 cm. Detailed parameters are displayed in Fig. 8.

**Figure 9:**
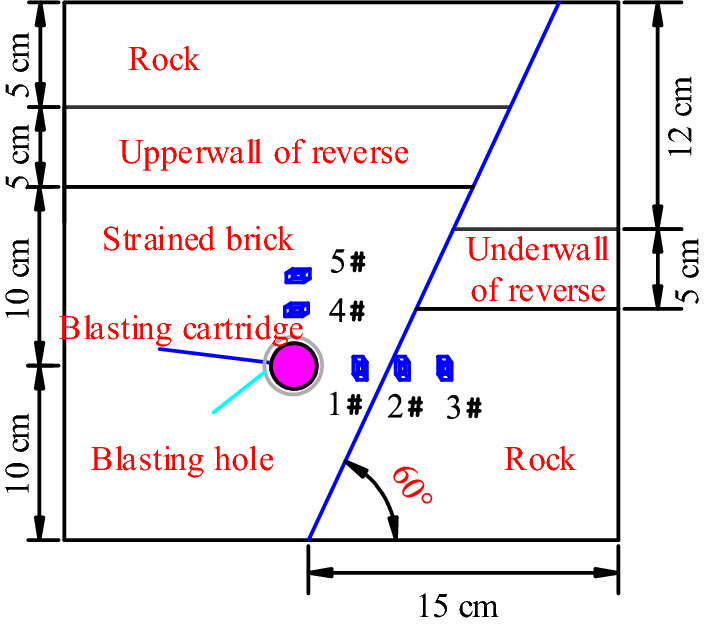
Stress measuring point.

Stress measuring points 4 and 5 were positioned directly above the blasting hole at vertical distances of 2 and 5 cm, respectively. Stress measuring points 1, 2, and 3 were arranged 2, 4, and 7 cm away, respectively, from the blasting hole in a horizontal direction (Fig. 9).

**Figure 10:**
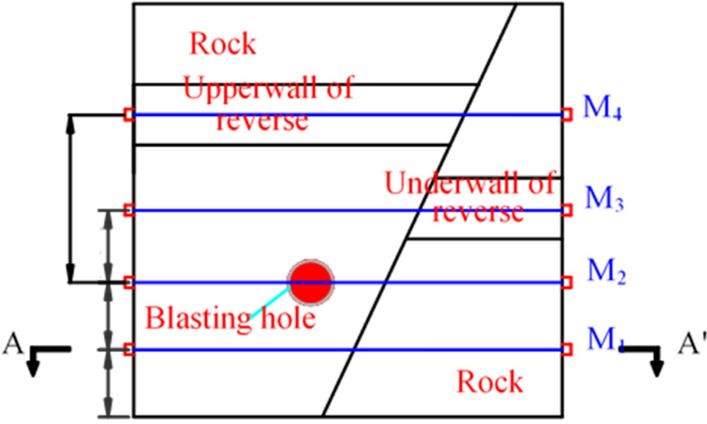
Surface layout for ultrasonic testing.

To measure the ultrasonic wave velocity of the experimental model after blasting and to assess the inversion of microcrack damage caused by the blasting load, sections M_1_, M_2_, M_3_, and M_4_ were selected for testing (Fig. 10). Section M_3_ passed through the lower wall of the reverse fault coal seam, whereas M_4_ passed through the upper wall of the reverse fault coal seam.

**Figure 11:**
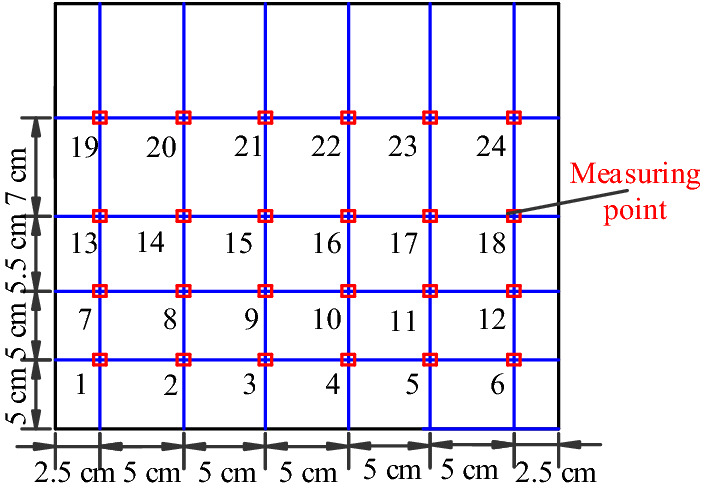
Ultrasonic testing point layout used in this study.

**Figure 12:**
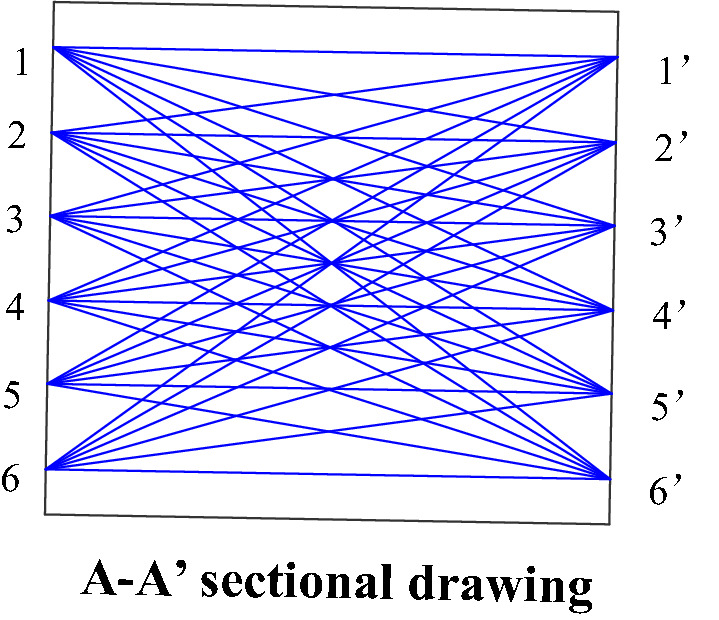
Diagram of launching and receiving ultrasonic test points.

Ultrasound transmitting and receiving points were arranged on two sides of the text block. Transmitting points were numbered from 1 to 24, whereas corresponding receiving points were numbered as 1′–24′ (Fig. 11). Six ultrasonic detection waves were emitted from one measuring point and were received at six corresponding sites on the other side of the same section (Fig. 12).

The experimental prototype was developed in the 14,136 working face of the 6th coal seam of Zhangji Mine within the Huainan Mining Area, Anhui Province, PR China, in this study. A total of 14 faults were developed in this working face, five of which had a drop greater than 3 m, and nine of which had a drop of less than 3 m. Original coal and rock mechanical parameters are outlined in Table 4.

**Table 4:** Summary of mudstone and coal seam mechanical parameters.

Rock type	Density (g/cm^3^)	Modulus of elasticity (GPa)	Poisson’s ratio	Compressive strength (MPa)	Tensile strength (MPa)
Mudstone	2.4	9.8	0.24	28.2	1.3
Coal seam	1.4	5.3	0.32	7.1	0.4

**Figure 13:**
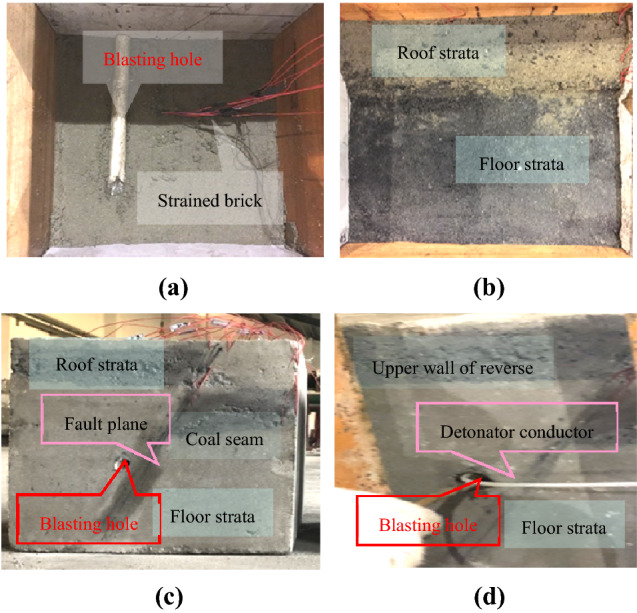
The entire process on the experimental model development: (**a**) rock layers of the reverse fault floor, (**b**) coal seam in the lower wall, (**c**) experimental model completion, and (**d**) charging and sealing.

The material ratio parameters of the experimental model are presented in Table 5. The experimental model was placed inside a prefabricated box. Strain bricks and reserved blasting holes were then embedded in their design position (Fig. 13a). In this case, the fault plane hardness was less than that of normal rock layers (Fig. 13b). Prepared samples were then air-dried at room temperature for a month (Fig. 13c). The samples were then loaded into the blasting simulation test device. The experimental blasting cartridge was then loaded into the hole, which was sealed with yellow mud (Fig. 13d).

**Table 5:** Material ratio parameters included in the blasting experiment for rock, fault plane, and coal seam.

Rock type	Cement	Coal	Gypsum	Sand	Water
Rock	1.2	0	0.5	6.1	0.70
Fault plane	1.0	0	0.8	6.2	0.80
Coal seam	0.2	1.8	1.2	2.5	0.65

**Figure 14:**
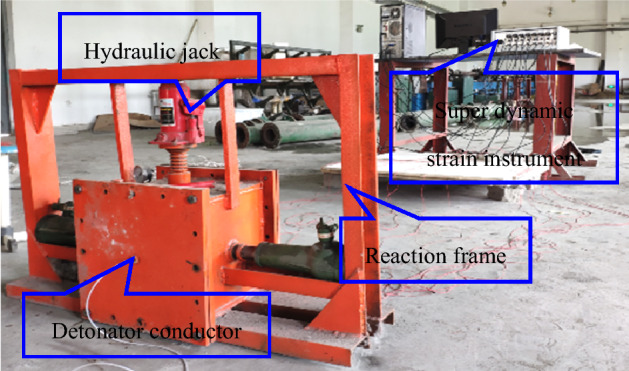
Blasting simulation with the experimental system.

Post to charge blasting, the experimental device was then filled with CO_2_ before the detonator and exploder were connected for blasting simulation tests after coal seam adsorption was balanced. The complete experimental system is depicted in Fig. 14.

### Analysis

**Figure 15:**
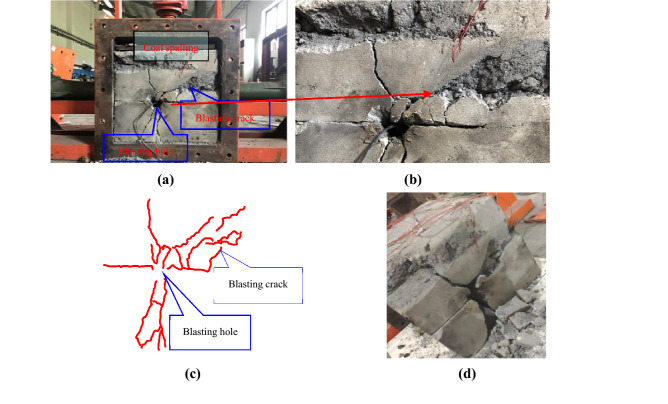
Crack development within the model after blasting: (**a**) development of cracks in the blasting model, (**b**) an enlarged partial map of the reverse fault structural belt, and (**c**) crack development on the surface of the blasting model, and (**d**) development of internal cracks in the blasting model.

Crack growth observed in the experimental model after blasting is displayed in Fig. 15. This figure shows that blasting cracks propagated around holes and subsequently generated crushing circles (Fig. 15a). The fault plane and floor rock adjacent to the footwall of the reverse fault were severely damaged in this experiment (Fig. 15b). The fracture extended to the upper and lower coal seams within the reverse fault. Furthermore, the surface body of the coal seam fell off, and internal damage was severe. A blasting fracture also developed, which produced an extensive crack between the coal seam fracture and the crushing circle around the blasting hole (Fig. 15c).

When the experimental model was removed from the blast simulation box, internal cracks were also developed in the sample (Fig. 15d). The upper and lower coal seam surfaces fell off the reverse fault, internal damage to the coal body was severe and cracks intersected with the crushing circle around the blasting hole.

**Figure 16:**
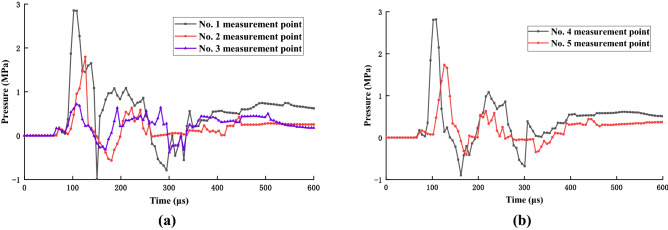
Blasting stress variation curves: (**a**) horizontal stress measurement points, and (**b**) vertical stress measurement points.

Stress curves for measuring points are displayed in Fig. 16. A positive value in this figure represents compressive stress generated by the blasting compressive wave, whereas a negative value represents tensile stress generated by the blasting tensile wave. This curve clearly revealed that peak stress decreased with an increase in the blasting hole distance but decreased with a decrease in time.

The stress similarity ratio, *K*_σ_, of the experimental model was 0.4, and its compressive and tensile strengths were 11.28 and 0.52 MPa. The compressive strength of the experimental model coal was 2.84 MPa, whereas the tensile strength was 0.16 MPa. Thus, when blasting stress was greater than the compressive strength of coal and rock, a blasting crushing circle was produced. Similarly, when blasting stress was greater than the tensile strength of coal and rock, blasting cracks were produced.

Data revealed that the stress recorded at measuring point 1 was the largest (2.9 MPa), which was greater than the tensile rock strength, resulting in radial blasting cracks. Because of the superposition of reflected tensile and incident stress waves at the coal–rock interface, multiple stress peaks were observed in this experiment. Thus, under coal seam influence in the lower wall of the reverse fault and the fault plane, the maximal stress at stress measuring point 2 could reach 1.8 MPa. Furthermore, the trend in stress change at measuring point 3 was similar to that at measuring point 2; here, the stress value was slightly less than that at measuring point 3, where the maximal value was 0.5 MPa (Fig. 16a).

Stress measurement points 4 and 5 were located far away from the coal seam on the upper wall of the reverse fault and were less affected by the fault plane. The maximal stress recorded at stress measuring point 4 was 2.8 MPa, whereas that at point 5 was 1.8 MPa. The recorded stress values at measuring points 4 and 5 were all greater than the tensile rock strength, which resulted in radial cracks (Fig. 16b).

**Figure 17:**
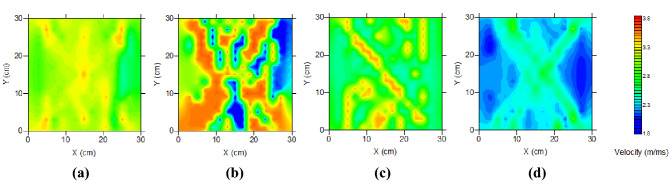
Ultrasonic image programs for different sections: (**a**) M_1_ section, (**b**) M_2_ section, (**c**) M_3_ section, and (**d**) M_4_ section.

The data cloud monitored by the ultrasonic detector post velocity value inversion is presented in Fig. 17. The Fig. 17a–d corresponds to the four planes of ultrasonic monitoring in Fig. 10. These data revealed that blasting cracks mainly developed toward the fault plane and coal seam within the lower wall of the reverse fault. These experimental results were consistent with those obtained from a numerical simulation.

## Mechanism analysis of outburst disturbance due to blasting

### Propagation rule for a blasting stress wave within a reverse fault tectonic zone

**Figure 18:**
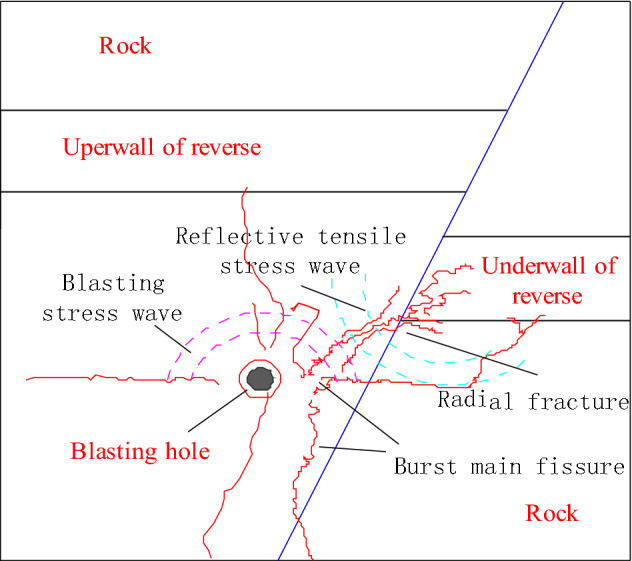
Schematic of the propagation of a blasting stress wave within a reverse fault.

The blasting stress wave propagation characteristics indicate that the wave impedance value for a coal seam is far less than that for a rock seam^20^. This indicates that when a blasting stress wave propagates to the coal seam within the lower wall of a reverse fault, a stress wave is transmitted and reflected at the coal–rock interface. This effect produces transmitted compression and reflected tension waves. Thus, compression and expansion deformations in coal and rock occur within an area where transmitted compression and reflected tension stress waves pass (Fig. 18)^21,22^.

Given the blasting impact load action, a stress wave was first caused by microdamage and microcracks in hard rock. The generation of tensile stress was closely related to the action of tensile waves in soft coal and fractured rock on fault planes and was concentrated in an area where wave impedance changed. Coal and rock were, therefore, both prone to tension–shear failure. Because of the reflection of stress waves adjacent to the coal–rock interface, the transformation of compressive stress and tensile stress were frequent, and energy was therefore held in a repeated cumulative state^23,24^. A transmitted compressive stress wave thus acted on the coal body and increased seam cracks. A reflected tensile stress wave then reacted on the rock and formed an extensive crack adjacent to the coal body, increasing damage and rock range.

### Analysis of coal and gas outbursts

Cumulative damage due to blasting vibration caused the most severe damage to the coal and rock interface adjacent to the reverse fault. Cross cracks near the interface between coal and rock formed a network crack; the larger the impact load was, the farther the altered distribution cracks would propagate inside the coal and rock to provide a passage for gas and form a weak surface for the occurrence of a dynamic disaster. These areas were, therefore, candidates for coal and gas outbursts.

Before a blasting stress wave was applied to the structure of coal and rock, free and adsorbed gases in coal pores and fissures remained in dynamic equilibrium. However, when a tensile stress wave acted on tectonic soft coal within the coal–rock interface area, a coal body in this area expanded and deformed, which resulted in an increase in pores and fissures. This destroyed the adsorption equilibrium state within coal as well as adsorbing gas dissolution^25^. Adsorbed free gas then diffused into the blasting crack area and caused an increase in the gas pressure within the coal seam, which provided a dynamic basis for the occurrence of coal and gas outbursts and increased the risk of these events.

The mechanical conditions leading to coal and gas outbursts mainly depended on tensile stress, gas pressure, and coal shear force strength. Penetrating cracks between coal and rock within the lower wall of reverse faults and blasting holes reduced the friction between coal internal bedding planes and their interfaces with the rock. Thus, when the tensile stress and gas pressure in coal were greater than the shear strength and friction force of coal and rock combined, gas internal energy stored in a coal seam and coal elastic potential were swiftly released along the weak broken surface between coal and rock, which resulted in outbursts.

## Conclusions

The following three conclusions were drawn:

- The propagation of blasting stress waves was highly influenced by reverse fault structures and fault planes, and the coal seam adjacent to the reserve fault under the wall adjacent to the hole was most strongly disturbed. A stress wave is transmitted and reflected when it propagated into a coal seam. Furthermore, the transformation of compressive and tensile stresses was frequent, which resulted in repeated accumulation of energy. This phenomenon indicated that damage to coal and rock in the underwall of a reverse fault was the most severe.
- A high concentration of stress and elastic strain energy accumulated within a reverse fault structural zone, which was a potentially dangerous area for coal and gas outbursts. A tensile wave formed by the reflection of the blasting stress wave acted on the rock between the hole and the underwall of the reverse fault, which reduced mechanical rock properties and provided a weak surface for outburst occurrence. A compression wave formed by the transmission of the blasting stress wave disturbed coal in the underwall of the reverse fault, increased cracks in the coal seam, adsorbed gas dissolution, and increased gas pressure. These factors created a dynamic basis for coal and gas outbursts.
- Blasting caused fragmentation of coal and rocks within a reverse fault tectonic region. This process attenuated the friction between the internal bedding planes of coal and the interface with the rock. Because tensile stress and gas pressure within a coal seam were greater than the shear strength and friction force of coal and rock, gas energy and the elastic potential of the seam were released swiftly along this weak surface, which resulted in coal and gas outbursts.
